# *Kalanchoe pinnata* Phytochemistry and Preclinical Pharmacology with an Appraisal of Safety and Clinical Evidence

**DOI:** 10.3390/molecules31193456

**Published:** 2026-09-28

**Authors:** Łukasz Kogut, Czesław Puchalski, Julia Jastrzębska, Grzegorz Zaguła

**Affiliations:** 1 Department of Bioenergetics, Food Analysis and Microbiology, Institute of Food Technology and Nutrition, Faculty of Technology and Life Science, University of Rzeszów, Aleja Rejtana 16C, 35-959 Rzeszow, Poland; lukasz.kogut2@wp.pl (Ł.K.); cpuchal@ur.edu.pl (C.P.); 2 Medical Centre in Łańcut LLC., Ignacego Paderewskiego 5, 37-100 Łancut, Poland; j.jastrzebska2105@gmail.com

**Keywords:** *Kalanchoe pinnata*, *Bryophyllum pinnatum*, phytochemicals, flavonoids, bufadienolides, wound healing, anti-inflammatory activity, antioxidant activity, antimicrobial activity, medicinal plants

## Abstract

**Background:** *Kalanchoe pinnata* (Lam.) Pers. is a medicinal species of the Crassulaceae family with a long history of ethnomedicinal use and a chemically diverse profile comprising flavonoids, bufadienolides, phenolic acids, triterpenes, and other secondary metabolites. Experimental studies have reported multiple biological effects of *K. pinnata* extracts and isolated constituents; however, the strength and clinical relevance of this evidence vary substantially among investigated applications. This review critically summarizes the phytochemical composition of *K. pinnata*, the proposed mechanisms underlying its biological activities, and the current level of experimental and clinical evidence relevant to its potential therapeutic use. **Methods:** A narrative review of the scientific literature was conducted using publications identified in PubMed/MEDLINE, Scopus, Web of Science, and Google Scholar. Studies addressing the phytochemical composition of *K. pinnata*, identification and characterization of its metabolites, biological mechanisms, pharmacological activity, safety, and available experimental and clinical evidence were considered. Particular attention was given to differences in study design, extract composition, experimental models, and the level of evidence supporting individual biological effects. **Results:** Phytochemical studies have identified flavonoids, bufadienolides, phenolic acids, triterpenes, and other secondary metabolites in *K. pinnata*, several of which have been associated with biological effects observed in experimental models. Quercetin glycosides, particularly quercetin 3-*O*-α-L-arabinopyranosyl-(1 → 2)-α-L-rhamnopyranoside (QAR), represent important bioactive constituents associated with anti-inflammatory, antioxidant, and wound-healing activities, whereas bufadienolides have demonstrated cytotoxic and antiproliferative effects in experimental cancer models but also represent an important toxicological concern. Preclinical evidence is most extensive for wound-healing, anti-inflammatory, antioxidant, antimicrobial, metabolic, and cytotoxic effects, whereas evidence for cardioprotective, hepatoprotective, nephroprotective, and other systemic applications remains predominantly experimental. However, differences in plant origin, phytochemical composition, extraction procedures, and experimental methodologies, together with the predominance of in vitro and animal studies, limit the direct translation of current findings into clinical practice. **Conclusions:** Current evidence supports the biological activity of several *Kalanchoe pinnata* extracts and isolated constituents under experimental conditions, but the extent to which these findings translate into clinically relevant therapeutic effects remains uncertain. The limited number of human studies, substantial variability in extract composition, incomplete pharmacokinetic characterization, and safety concerns related particularly to bufadienolides currently restrict broader clinical interpretation. Further research should prioritize standardized preparations, clearly characterized phytochemical profiles, dose–response relationships, long-term safety assessment, and well-designed controlled clinical trials.

## 1. Introduction

### 1.1. Species Characteristics

*Kalanchoe pinnata* (Lam.) Pers., also known by the taxonomic synonyms *Bryophyllum pinnatum* and *Bryophyllum calycinum*, is a perennial succulent belonging to the family Crassulaceae [1,2,3,4]. The plant usually reaches a height of 0.3–1.5 m, although under favorable environmental conditions it may grow up to approximately 2 m [1,2,3,5,6]. It develops tall, branched, hollow stems that are reddish and covered with numerous white spots in young plants, becoming lighter, smoother, and more lignified with age [2,3,5,7].

The leaves of *Kalanchoe pinnata* are fleshy, dark green, and oppositely arranged [3,6,7,8,9]. Their morphology changes as the plant develops [3,10]. Leaves located on the lower parts of the shoots are usually simple, whereas those in the upper parts become pinnately compound and typically consist of 3–7 elliptical or ovate leaflets [3,6,9,11]. A characteristic feature is the distinctly crenate–serrate leaf margin, which is often bordered by purple or reddish pigmentation [3,6,9,12]. Anatomically, the leaves contain anisocytic stomata and are glabrous on both the adaxial and abaxial surfaces [2,5,9,11].

The most distinctive biological feature of this species is its highly developed capacity for vegetative reproduction [4,13,14]. Numerous plantlets (bulbils) develop in notches along the leaf margins and, after detaching from the mother plant and coming into contact with the substrate, rapidly develop roots and give rise to new individuals [1,2,6,7,13]. This characteristic underlies the Polish common name “żyworódka” and contributes to the species’ ability to spread readily both in natural environments and under cultivation [14,15].

The flowers of *K. pinnata* are pendulous and bell-shaped and are arranged in large terminal panicles [3,5,6,7,8,9]. They are characterized by a tubular calyx, distinctly inflated at the base and brownish-purple in color, together with a reddish-purple corolla [3,5,6]. *K. pinnata* is cultivated as an ornamental plant and is also used as a medicinal plant material in traditional and phytotherapeutic applications [5,7,16,17,18].

### 1.2. Distribution and Use in Traditional Medicine

*Kalanchoe pinnata* is native to Madagascar; however, owing to its high adaptive capacity, the species has spread extensively throughout tropical and subtropical regions [1,3,4,19,20,21]. It is now widely distributed across many regions of Asia, Africa, the Americas, and Australia, as well as numerous Pacific islands. Its ease of propagation and low habitat requirements have contributed to its widespread establishment beyond its native range [1,4,21,22,23].

For centuries, *K. pinnata* has occupied an important place in the traditional medicine of various regions worldwide [1,24,25,26,27,28,29]. Owing to its extensive ethnomedicinal use, *K. pinnata* is commonly referred to in the literature as the “miracle leaf” [4,19,28,30,31]. Traditionally, it has been used primarily for the treatment of difficult-to-heal wounds and skin injuries, as well as ear infections, respiratory disorders, fever, gastric ulcers, kidney stones, and various inflammatory conditions [1,2,5,23,26,32]. These ethnomedicinal uses have provided a rationale for subsequent phytochemical and pharmacological investigations of *Kalanchoe pinnata* and its constituents [4,7,8,25,28,31].

### 1.3. Phytotherapeutic Significance

Phytochemical studies of *Kalanchoe pinnata* have identified several groups of secondary metabolites, including flavonoids, bufadienolides, phenolic acids, and triterpenes [8,22,33,34,35,36]. Extracts containing these compounds, as well as selected isolated constituents, have been investigated in experimental studies for their effects on inflammation, tissue repair, microbial growth, glucose metabolism, and cell proliferation [7,8,28,31].

Wound healing is among the more extensively investigated biological effects of *K. pinnata* [4,5,7,37,38]. In experimental wound models, leaf extracts have been associated with improved re-epithelialization and changes in collagen deposition and organization [7,20,36,37,39,40,41]. QAR, one of the major flavonoids identified in the plant, has also been investigated in this context and has been proposed as a bioactivity marker of *Kalanchoe pinnata* preparations [3,14,20,33,42].

Anti-inflammatory and analgesic effects of *K. pinnata* have also been reported in experimental studies [1,15,43]. Proposed mechanisms include inhibition of cyclooxygenase activity and modulation of inflammatory mediators, including TNF-α, prostaglandins, and leukotrienes [4,29,43,44]. These observations are consistent with the traditional use of *K. pinnata* in inflammatory conditions, although evidence supporting specific clinical indications remains limited [14,21,31,45].

Bufadienolides and other *Kalanchoe pinnata* constituents have been discussed as candidates for experimental cytotoxicity research [46,47]. This interpretation requires separation of isolated compounds from extracts and comparison with nonmalignant cells. Current evidence does not establish anticancer efficacy in humans.

Metabolic effects of *Kalanchoe pinnata* have been investigated mainly in experimental models. A leaf-derived dichloromethane fraction has been studied for insulin secretion and glucose outcomes in diabetic rats [48]. An aqueous extract has also been tested in salt-loaded hypertensive rats [26]. These findings do not establish clinical antidiabetic or antihypertensive efficacy.

Renal and hepatic effects of *Kalanchoe pinnata* have been investigated in animal models of chemically induced injury. An aqueous leaf extract reduced biochemical and histological renal injury during gentamicin exposure [49], whereas leaf juice and an ethanolic leaf extract were examined in carbon tetrachloride-induced liver injury [50]. These findings do not establish organ-protective efficacy in humans. Research on *Kalanchoe integra var. crenata* is comparative evidence and is not used to support these species-specific statements [51].

Human investigations of *Kalanchoe pinnata*, frequently published under the synonym *Bryophyllum pinnatum*, include preterm labour and bladder symptoms [52,53,54,55]. Different formulations and study designs must be considered separately: retrospective comparisons and small or discontinued trials do not establish general tocolytic or urological efficacy.

Antimicrobial investigations of *Kalanchoe pinnata* include defined leaf fractions tested against bacteria [29] and root-derived compounds evaluated in antiviral assays [56]. These are preparation-specific in vitro findings; they do not establish efficacy against clinical infection.

Previous reviews have described ethnomedicinal uses and phytochemistry [3,4,6], clinical use of *Bryophyllum* preparations [13], bufadienolides [47], and proposed anticancer or antidiabetic applications [24,46]. The remaining issue addressed here is the traceability of biological claims to the species, preparation, and level of evidence actually investigated. This narrative synthesis therefore integrates chemical identification confidence, experimental pharmacology, preparation-specific safety, and the limited human literature. Its contribution is a critical assessment of the barriers to clinical translation, including null clinical findings and unresolved exposure–toxicity relationships, rather than a claim to discover new constituents or establish therapeutic efficacy.

## 2. Materials and Methods

This article is a critical narrative review of *Kalanchoe pinnata* (Lam.) Pers. across phytochemistry, preclinical pharmacology, safety, and limited clinical investigation. A narrative design was chosen because the objective is to interpret heterogeneous preparations and evidence types across several biological questions, rather than estimate a pooled treatment effect for a single indication. Literature was identified using PubMed/MEDLINE, Scopus, Web of Science, and Google Scholar, with an emphasis on publications from 2000 to 2026; earlier primary studies were retained where relevant. Searches included the species name and the synonyms *Bryophyllum pinnatum* and *Bryophyllum calycinum*, combined with terms concerning phytochemistry, extraction, analytical characterization, biological activity, mechanisms, toxicity, and clinical use. Reference lists provided additional sources. This description represents a topic-guided literature search and does not claim an exhaustive systematic search or compliance with PRISMA 2020.

English-language full-text publications relevant to the review questions were considered. Primary studies support preparation-specific quantitative, mechanistic, and clinical statements; reviews provide background and help locate original reports. Abstract-only reports and secondary summaries are not treated as independently verified full-text evidence. Species-specific synthesis includes studies explicitly identifying *Kalanchoe pinnata* or its stated taxonomic synonyms, and multispecies studies only where results for this species can be separated. Work on other *Kalanchoe* species is used solely as labeled comparative context, to illustrate chemical variability or generate mechanistic and safety hypotheses. It is excluded from estimates of *Kalanchoe pinnata* composition, potency, clinical efficacy, and exposure safety. Transgenic preparations and multicomponent formulations are distinguished from native, single-species preparations.

The synthesis distinguishes isolated compounds, chemically characterized fractions, crude extracts, and formulated products. Chemical identification is separated from quantification, and biological findings are classified as cell-free assays, cellular or ex vivo experiments, animal outcomes, or clinical observations. Computational predictions are discussed separately from experimentally measured mechanisms. Quantitative examples were chosen to illustrate defined preparations and measurable endpoints across the main experimental domains; they are not an exhaustive effectiveness dataset. The human evidence distinguishes randomization, comparators, exposure, outcomes, adverse events, and design limitations. Available negative or null findings are retained. Study limitations are appraised qualitatively, including controls, sample size, randomization and blinding when documented, attrition, chemical characterization, and selective reporting. No pooled effect estimate or validated study-level risk-of-bias instrument was applied; consequently, no formal certainty grading or low-risk-of-bias designation is assigned.

The search and selection process is not fully reproducible from the available reporting: database-specific search histories, record counts at successive selection stages, the final search date, and the extent of Google Scholar screening are not documented. Independent duplicate screening and data extraction are likewise not established by the reported methods. These limitations prevent reconstruction of a numerical selection flow and increase the possibility of selection, language, and publication bias. The review should therefore be interpreted as a critical, non-exhaustive narrative synthesis.

As this study constitutes a narrative review of the literature and did not involve human or animal participants, the use of personal data, or experimental procedures, approval by a bioethics committee and study registration were not required.

## 3. Phytochemical Profile and Bioactive Compounds of *Kalanchoe pinnata*

### 3.1. Flavonoids

The phytochemical literature reports flavonoids, bufadienolides, phenolic acids, triterpenes, and other metabolites in *Kalanchoe pinnata* [3,4,6]. These reports combine findings from different plant parts and preparations and encompass isolated compounds, reference-supported identifications, and tentative mass-spectrometric annotations [29,57]. They should not be interpreted as a count of structurally confirmed compounds present together in a single extract. A species-wide total is therefore not assigned here. However, the qualitative and quantitative composition of these metabolites is not constant and depends on numerous factors, including the geographical origin of the plant, leaf age, developmental stage, and environmental conditions, particularly light exposure [23,57,58]. This variability affects both the concentrations of individual compounds and the biological activity of the resulting extracts [10,57].

Flavonoids are among the most extensively characterized groups of secondary metabolites in *Kalanchoe pinnata* [14,23,42]. In a methanolic leaf extract, flavonoid glycosides were the main UV-absorbing constituents detected by HPLC-PDA-ESIMS [57]. This describes the detector response under the reported analytical conditions, not their percentage by mass in the extract. Relative peak areas depend on the detector, wavelength or ionization response and cannot establish concentration, molar abundance, or biological importance without appropriate calibration. Among these, flavonol glycosides based primarily on quercetin and kaempferol aglycones predominate [7,14,59]. The glycosylation pattern of these compounds can influence their solubility, chemical stability, and biological activity [60].

The level of structural evidence must be specified separately from quantification. Fürer et al. isolated nine flavonoids and two phenolic glucosides from a methanolic leaf extract and established their structures by 1H and two-dimensional NMR; four bufadienolides were detected using chromatographic and mass-spectrometric data with authentic reference compounds [57]. By contrast, LC-QTOF-MS profiling of an aqueous fraction derived from an ethanolic leaf extract included tentative assignments for which exact sugar identity or substitution pattern was not always established [29]. Such annotations are distinguished from isolated, structurally confirmed compounds. Semi-quantitative estimates obtained with related standards likewise do not constitute compound-specific absolute quantification [29].

Quercetin 3-*O*-α-L-arabinopyranosyl-(1 → 2)-α-L-rhamnopyranoside (QAR) is one of the characteristic flavonoids identified in *K. pinnata* and has been proposed as a chemical marker for the standardization of plant preparations [14,33,42,61]. QAR is a quercetin diglycoside with an uncommon glycosylation pattern [60]. Experimental studies have investigated this compound in relation to anti-inflammatory, antioxidant, and gastroprotective effects [14,20,33,42].

In addition to QAR, numerous other quercetin derivatives have been reported in the leaves and flowers [4,13,44]. These include quercitrin (quercetin 3-*O*-rhamnoside), rutin, isoquercitrin, and miquelianin (quercetin 3-*O*-glucuronide) [4,14,31,44,62]. A QAR isomer, quercetin 3-O-β-D-xylopyranosyl-(1 → 2)-α-L-rhamnopyranoside, has also been identified in *K. pinnata* [4,14,33,57].

The second major group comprises kaempferol derivatives [4,6,8,10]. The most characteristic representative is kapinnatoside, identified as kaempferol 3-*O*-α-L-arabinopyranosyl-(1 → 2)-α-L-rhamnopyranoside [6,14]. Similarly to QAR, it possesses an unusual arrangement of sugar residues and is considered a characteristic compound of *K. pinnata* [14,61]. Other identified kaempferol derivatives include afzelin, astragalin, and kaempferitrin [6,17,31,42].

The flavonoid profile of the plant is further complemented by several less abundant compounds belonging to other flavonoid subclasses [6,10,42]. Literature reports include, among others, 4′,5-dihydroxy-3′,8-dimethoxyflavone 7-*O*-β-D-glucopyranoside, as well as myricetin derivatives such as myricitrin and compounds related to luteolin, eupafolin, mearnsetin, and gossypetin [6,18,29,57,63].

For flower preparations, Villarreal Romero et al. used a hot-water infusion and purified QAR and quercetin 3-O-D-glucuronide by centrifugal partition chromatography; the broader flavonoid profile was assigned using UV and mass-spectrometric information [63]. Their validated UPLC-PDA quantification and the assigned chromatographic profile are distinct analytical outputs and apply to the investigated flower extract, not to all leaf or whole-plant preparations [63].

### 3.2. Bufadienolides

Another key group of secondary metabolites occurring in *Kalanchoe pinnata* comprises bufadienolides [6,10,31,61]. These are C-24 steroids containing a characteristic six-membered lactone ring attached at the C-17β position [14,42]. Bufadienolides are pharmacologically active compounds that are also relevant to the toxicological profile of *K. pinnata* [24,42,47]. Selected bufadienolides isolated from *Kalanchoe pinnata* have demonstrated cytotoxic and antiproliferative effects in experimental cancer models [28,46,47].

Bufadienolides reported for *Kalanchoe pinnata* include bryophyllins and bersaldegenin derivatives [13,47]. In the primary methanolic leaf-profile study, bryophyllin A and bersaldegenin-1-acetate, -3-acetate, and -1,3,5-orthoacetate were detected with authentic reference compounds [57]. Their occurrence does not demonstrate that all have the same potency or mechanism in an uncharacterized preparation.

The bufadienolide content varies according to the developmental stage of the plant [6,10,13]. Higher concentrations have been reported in young leaves, with lower levels observed in older plant material [6,13,24]. Owing to their lipophilic properties, bufadienolides are commonly isolated using organic solvents such as dichloromethane [29,42,47,57]. Their limited water solubility may reduce their extraction into aqueous preparations compared with organic solvent extracts [10,42,57,60].

### 3.3. Phenolic Acids

Phenolic acids constitute another important component of the phytochemical profile of *Kalanchoe pinnata* [19,23,24,29]. They occur primarily as derivatives of benzoic and cinnamic acids and, together with other phenolic constituents, may contribute to the antioxidant properties of the plant [6,8,29,41].

Numerous representatives of this group have been reported in leaf extracts, including gallic, caffeic, ferulic, syringic, protocatechuic, p-coumaric, and vanillic acids [4,6,18,19,41,64]. In addition to free phenolic acids, their esters and glycosides have also been detected [3,13,57]. Among the characterized conjugated derivatives are syringic acid β-D-glucopyranosyl ester and 4′-O-β-D-glucopyranosyl-cis-p-coumaric acid [3,6]. The presence of both free and conjugated phenolic acids contributes to the chemical diversity of the phenolic fraction of *K. pinnata* [29,58].

### 3.4. Triterpenes and Sterols

Triterpenes and phytosterols have been reported in *Kalanchoe pinnata*, although many species-wide lists are compiled in secondary reviews and do not provide preparation-specific concentrations [3,4,6]. Isolation from a related species does not establish occurrence or abundance in *Kalanchoe pinnata*.

Review-level reports include α-amyrin, β-amyrin, and additional triterpenes [3,4,6]. These inventories should be distinguished from primary structural confirmation in an authenticated sample. No ranking of predominant triterpenes or concentration in a typical preparation is assigned on the basis of these reports.

Sterols are also included in secondary accounts of *K. pinnata* chemistry [3,6]. The isolation of β-sitosterol from *Kalanchoe tomentosa* [65] and chemical profiling of *Kalanchoe fedtschenkoi* [66] are not species-specific confirmation for *K. pinnata*. Representative primary analytical findings are therefore prioritized in Table 1.

### 3.5. Other Constituents and Primary Metabolites

In addition to the major classes of secondary metabolites, *Kalanchoe pinnata* contains several other groups of phytochemicals [6,15,28]. Qualitative phytochemical screening has reported alkaloid- and saponin-positive reactions in leaves and stems [6,8,11,67]. These class-level tests do not establish individual molecular structures or concentrations. Compared with flavonoids and bufadienolides, these constituents have been less extensively characterized in *Kalanchoe pinnata* [15,39,41].

Organic acids constitute another component of the chemical profile of *K. pinnata* and are associated with Crassulacean Acid Metabolism (CAM) [10,20]. Citric, isocitric, malic, oxalic, succinic, and oxaloacetic acids have been identified in plant tissues [6,17,21,28]. Their occurrence is consistent with the CAM characteristic of many succulent species of the family Crassulaceae [10,20,42].

Fatty acids, amino acids, carbohydrates, organic acids, and vitamins belong to broader nutritional and metabolic components of the plant and are not collectively classified here as secondary metabolites. Minerals are elements rather than metabolites. Secondary accounts describe these constituents in *Kalanchoe pinnata* [6,31], but qualitative detection does not establish nutritional adequacy, high abundance, or therapeutic relevance at an administered dose.

Phenanthrene describes a carbon-ring scaffold and does not, by itself, identify an alkaloid. In the absence of a named, structurally characterized nitrogen-containing compound, reports of phenanthrene derivatives are not classified here as alkaloids. The nitrile glycoside sarmentosin was tentatively annotated in the leaf-derived aqueous fraction [29]; an LC-MS annotation or class-positive screening reaction is distinct from isolation and full structural elucidation.

Table 1 summarizes representative phytochemical findings with the plant part, extraction solvent or fractionation procedure, and level of analytical evidence. The entries describe specified preparations and do not imply that every reported constituent occurs in all *K. pinnata* extracts.

Figure 1 summarizes representative phytochemical evidence and the limits of its interpretation.

The scheme distinguishes structural confirmation from tentative annotation. Chemical identification alone neither establishes an effective dose nor identifies the constituent responsible for an extract-level biological effect.

### 3.6. Preliminary Structure–Activity Relationships of Selected Phytochemicals

Although the available data do not permit the establishment of a comprehensive structure–activity relationship (SAR) for the phytochemicals of *Kalanchoe pinnata*, comparison of structurally related compounds provides several preliminary observations. These relationships are particularly evident among bufadienolides, whereas for flavonoids the available evidence is less specific and should be interpreted cautiously [42,47].

Bufadienolides possess a steroidal scaffold with a six-membered α-pyrone ring at C-17. Substitutions, including hydroxylation, acetylation, and orthoacetate formation, can influence biological activity [47]. Reports of inhibition of Epstein–Barr virus early-antigen activation concern a tumor-promotion assay; they are not equivalent to antiviral replication inhibition or clinical anticancer efficacy. Comparisons summarized in genus-level reviews are preliminary structure–activity hypotheses unless the compound identity, source species, assay, and comparator conditions are matched.

For flavonoids, structural differences involving the aglycone and the number, identity, and position of sugar residues may influence physicochemical properties and biological activity. *K. pinnata* contains numerous quercetin- and kaempferol-based glycosides, including QAR, quercitrin, rutin, isoquercitrin, and kapinnatoside [4,14,42]. Glycosylation can modify molecular polarity, solubility, chemical stability, membrane permeability, and susceptibility to enzymatic hydrolysis and may therefore influence the biological availability and activity of individual flavonoid derivatives [42,60]. QAR is particularly relevant because its quercetin nucleus is substituted with an unusual arabinose–rhamnose disaccharide [42,57]. Experimental studies have associated QAR with anti-inflammatory and antinociceptive effects [44], as well as wound-healing activity [37]. However, direct comparative studies of QAR and closely related *K. pinnata* flavonoids under identical experimental conditions remain limited, precluding firm conclusions regarding which individual structural features are responsible for these activities.

Overall, the available evidence indicates that relatively small structural differences within the major phytochemical classes of *K. pinnata* may influence biological activity. The clearest preliminary relationship concerns bufadienolides, for which the steroidal scaffold, C-17 α-pyrone ring, and substitution pattern, particularly the presence of an orthoacetate moiety, appear relevant to biological potency [47]. In contrast, SAR interpretation for flavonoid glycosides remains largely inferential because systematic head-to-head comparisons are lacking. Further studies using structurally related isolated compounds evaluated under standardized experimental conditions are required to establish more definitive SAR models.

## 4. Preclinical Pharmacology and Limited Clinical Evidence

Studies of *Kalanchoe pinnata* have reported biological effects involving inflammatory, oxidative, metabolic, immunological, regenerative, and proliferative processes [14,24]. The available evidence includes studies of wound healing, inflammation and nociception, antioxidant activity, microbial and parasitic growth, glucose metabolism, cardiovascular, hepatic and renal function, smooth muscle contractility, and cancer cell proliferation [7,19,24]. These effects have been investigated using both *Kalanchoe pinnata* extracts and isolated constituents, particularly flavonoids and bufadienolides [14]. However, the level of evidence differs substantially among these areas, and many of the reported effects are based primarily on in vitro experiments or animal models.

Biological effects are attributed below to the material actually tested: crude extract, enriched fraction, formulated product, or isolated compound. Detection of a constituent in an active extract does not demonstrate that it caused the extract-level effect, and proposed contributions or mechanisms require direct testing. Human-cell cultures and isolated human tissues remain in vitro or ex vivo evidence, respectively, rather than clinical studies in treated participants.

### 4.1. Wound Healing and Tissue Regeneration

Wound healing is one of the more extensively investigated biological effects of *Kalanchoe pinnata* [5,28]. Experimental studies have associated *Kalanchoe pinnata* preparations with changes in several processes involved in tissue repair, including inflammatory responses, collagen deposition, vascularization, and re-epithelialization [20,28]. Effects on wound closure, granulation tissue formation, and extracellular matrix remodeling have also been reported in experimental wound models [20].

In rat excision wounds, creams containing 6% aqueous *Kalanchoe pinnata* leaf extract or 0.15% purified QAR were associated with more advanced re-epithelialization and denser collagen organization [37]. These histological observations concern topical treatment in animals. Hydroxyproline measurement in plant material is not a measure of collagen deposition in a treated wound, and wound-healing experiments with other species are not used as evidence for *K. pinnata*.

Angiogenesis has also been investigated as a possible component of the wound-healing response to *K. pinnata* preparations. In an experimental wound model, application of a gel containing plant-derived constituents was associated with increased expression of vascular endothelial growth factor (VEGF) [20]. Because VEGF promotes new blood vessel formation, increased expression of this factor may contribute to vascularization and granulation tissue formation during wound repair [20,33].

In rat excision wound models, *Kalanchoe pinnata*-based preparations have been associated with increased wound closure [20,37]. In treated groups, wound healing rates on day 12 ranged from 95.3% to 97.5% [14,37]. In the reported experimental model, these outcomes were comparable to those observed with reference preparations containing neomycin and bacitracin [37]. Together with the histological findings described above, these results indicate that *K. pinnata* preparations can influence several components of tissue repair under experimental conditions [28,37].

Direct human evidence for wound care includes a quasi-experimental pretest–post-test controlled study of 40 patients with diabetic foot ulcers, allocated to leaf-decoction cleansing or 0.9% saline cleansing (20 per group) [68]. The intervention used 100 g of leaves boiled in 1 L of water to approximately 500 mL, with six treatments over 14 days. Both groups improved; the authors reported greater improvement with the decoction (between-group *p* = 0.003), although baseline differences and unclear reporting of the between-group test across individual wound indicators limit interpretation. This was a study of wound cleansing with an aqueous decoction, not a case–control study of leaf dressings. Its short duration and design do not establish complete ulcer healing, prevention of amputation, or efficacy of isolated QAR. Study details and limitations are summarized in Table 3 [68].

### 4.2. Anti-Inflammatory and Analgesic Activities

Anti-inflammatory effects of *Kalanchoe pinnata* have been reported in experimental studies using plant extracts and isolated constituents [5,20,28]. The investigated mechanisms include effects on enzymes involved in the synthesis of inflammatory mediators, prostaglandin production, cytokine signaling, and immune cell activity [24,44].

In biochemical assays, the isolated *Kalanchoe pinnata* flavonoid QAR, designated KPFV in the study, inhibited COX-1 with IC_50_ = 22.1 µg/mL; the reported COX-2 IC_50_ exceeded 50 µg/mL [44]. This is direct enzyme-assay evidence for the isolated compound. It does not show that an unspecified extract achieves the same target inhibition or that these concentrations occur in humans.

Modulation of cytokine production has also been reported following exposure to *K. pinnata* preparations [24,43]. Experimental studies have described reductions in pro-inflammatory mediators, including TNF-α, IL-1β, and IL-6, together with increased levels of the anti-inflammatory cytokine IL-10 [24,43]. Because these mediators participate in the initiation, maintenance, and resolution of inflammatory responses, the observed changes suggest that *K. pinnata* preparations may influence both pro- and anti-inflammatory signaling pathways [24,43].

Antinociceptive effects have also been reported in experimental models [7,44,62,69]. Subcutaneous administration of *K. pinnata* flower extract at doses ranging from 30 to 300 mg kg^−1^ inhibited acetic acid-induced nociceptive responses in mice, with a reported ID50 value of 164.8 mg kg^−1^ [44]. Experimental findings suggest that this activity may involve both peripheral mechanisms associated with modulation of pain-related mediators and central mechanisms affecting nociceptive signal transmission [3,7]. However, these findings are derived from animal models and do not establish analgesic efficacy in humans.

Effects of *K. pinnata* constituents on mast cell-mediated responses have also been investigated experimentally [7,20,45]. Plant-derived constituents have been reported to inhibit antigen-induced mast cell degranulation [7,45,62]. Because mast cell degranulation leads to the release of mediators involved in edema, smooth muscle contraction, and amplification of inflammatory responses, this mechanism may contribute to the anti-inflammatory and immunomodulatory effects observed in experimental models [20,70]. However, the available evidence does not establish that *K. pinnata* preparations prevent anaphylaxis or provide clinically effective treatment for allergic airway diseases.

### 4.3. Antioxidant Activity

Antioxidant evidence for *Kalanchoe pinnata* must be separated into chemical radical-scavenging or reducing capacity, cellular responses, changes in animal oxidative-stress biomarkers, and clinically meaningful effects. DPPH and ABTS are cell-free assays; neither independently demonstrates antioxidant efficacy in animals or humans. Changes in enzyme activities or glutathione in animal tissues remain model-specific biomarkers rather than proof of a clinical benefit [26,49].

In DPPH and ABTS radical-scavenging assays, *Kalanchoe pinnata* extracts demonstrated activity that varied with the preparation and assay [24,27,39,71]. Harlalka et al. reported a DPPH EC50 of 116.25 µg mL^−1^ for an aqueous leaf extract prepared by cold maceration [49]. The assay used methanolic DPPH (0.1 mM), extract solutions of 50–250 µg mL^−1^, a 30 min incubation at 25 °C, and absorbance measurement at 517 nm; EC50 represented the concentration required to scavenge 50% of DPPH radicals under those conditions [49]. This preparation-specific chemical-assay value cannot be ranked directly against values for different solvents, fractions, plant parts, radical systems, or incubation protocols. Although phenolic constituents may contribute, the activity of the crude extract cannot be assigned quantitatively to individual flavonoids or phenolic acids without direct comparative experiments. These cell-free results do not establish antioxidant efficacy in humans.

Changes in antioxidant enzymes and glutathione reported in animal models should be interpreted alongside the organ-injury model, preparation, dose, and route [26,49]. Such biomarkers cannot be treated as equivalent to chemical scavenging values, direct molecular target engagement, or patient outcomes.

Metal-ion chelation has been proposed as an additional mechanism contributing to the antioxidant activity of *K. pinnata* constituents [25,39,72]. Because free iron can participate in reactions that generate reactive species and initiate lipid peroxidation, its chelation may limit iron-dependent oxidative reactions [24,39,72]. This mechanism has been investigated experimentally, but its contribution to the antioxidant effects of *K. pinnata* in vivo remains insufficiently characterized [28,39].

### 4.4. Antimicrobial and Antiparasitic Activities

Antimicrobial activity of *Kalanchoe pinnata* is interpreted at the level of the tested preparation, microbial strain, and assay. Growth inhibition alone does not demonstrate membrane disruption or inhibition of protein synthesis. Transgenic plants expressing cecropin P1 constitute a separate experimental intervention [36,38].

In a broth-microdilution study, the aqueous fraction obtained by partitioning a 95% ethanolic leaf extract of *Kalanchoe pinnata* inhibited *Staphylococcus aureus* ATCC 6538 and *Staphylococcus epidermidis* ATCC 14990 at MICs of 64 and 32 µg/mL, respectively; MBCs exceeded 2000 µg/mL for both strains [29]. Ampicillin MICs were 0.08 and 0.313 µg/mL, respectively. For *Candida albicans* ATCC 10231, the same aqueous fraction had MIC and MBC values above 2000 µg/mL. These findings distinguish inhibitory activity from bactericidal activity and retain an inactive fungal result. They do not establish activity across the ESKAPE group. CC_50_ and antimicrobial selectivity indices were not established for these preparations in matched nonmalignant-cell assays [29].

Flavonoids isolated from *Kalanchoe pinnata* have been evaluated against *Leishmania amazonensis* in experimental antileishmanial assays [61]. These data do not support a general claim that plant preparations are as effective as meglumine antimoniate with lower toxicity. Such a comparison would require the same model, parasite stage, exposure, comparator regimen, and measured toxicity endpoints. The present synthesis therefore does not assign comparative clinical efficacy or safety to these preparations.

Human antileishmanial evidence is limited to an uncontrolled report of one adult treated orally with 30 g fresh leaves per day for 14 days. The lesion stopped enlarging and decreased slightly, but clinical cure was not demonstrated; the observation cannot establish efficacy or general safety [73].

Cryer et al. isolated root-derived compounds and characterized them by MS and NMR [56]. In Vero-76 cells, KPB-100 inhibited HSV-2 E194, thymidine-kinase-deficient isolate 1253, and isolate 10,161 with reported IC_50_ values of 5.4, 4.6, and 4.5 µg/mL, respectively; CC_50_ exceeded 25 µg/mL, giving reported selectivity indices above 4.6, 5.4, and 5.6. Acyclovir and cidofovir were parallel controls. The methods describe cytopathic-effect inhibition, despite the table caption referring to plaque reduction. KPB-100 also inhibited HSV-1 v2424 virus yield, with IC_90_ = 3.0 µg/mL; IC_90_ is not interchangeable with IC_50_. These functional antiviral results do not identify a molecular target and do not establish efficacy of crude leaf preparations [56].

### 4.5. Antidiabetic Potential

The effects of *Kalanchoe pinnata* on glucose metabolism have been investigated primarily in experimental models [6,24,74]. Reported mechanisms include inhibition of carbohydrate-digesting enzymes, modulation of insulin secretion, effects on cellular glucose uptake, and changes in signaling pathways involved in energy metabolism [6,24,74]. However, evidence supporting these effects is predominantly preclinical, and their relevance to glycemic control in humans remains uncertain.

Inhibition of carbohydrate-digesting enzymes has been investigated as one possible mechanism underlying the metabolic effects of *K. pinnata* [6,24,74]. In enzymatic assays, *K. pinnata* extracts inhibited α-amylase and α-glucosidase, with reported IC50 values of 29.50 µg mL^−1^ and 32.04 µg mL^−1^, respectively [24,74]. Because these enzymes participate in the digestion of carbohydrates and subsequent release of absorbable glucose, their inhibition could theoretically reduce the rate of postprandial glucose availability [48,74]. However, enzyme inhibition observed in vitro does not by itself establish a glucose-lowering effect in humans.

A dichloromethane fraction of *Kalanchoe pinnata* leaf steam distillate stimulated insulin secretion in an experimental assay [48]. The primary abstract reports a 3.29-fold increase at 10 µg/mL relative to a positive control. The concentration is 10 µg/mL, not 10 mg/mL. Because the complete comparator and secretion dataset are not resolved here, the fold-change is not used to claim superiority to a named antidiabetic drug. Pharmacological evidence of K-ATP-channel involvement is distinct from direct binding to the channel, and findings for this fraction cannot be generalized to all plant extracts.

Effects on peripheral glucose metabolism have also been investigated [24]. Network-pharmacology and molecular-docking analyses have proposed targets related to insulin and PI3K/Akt signaling [74]. Such predictions generate hypotheses but do not demonstrate pathway modulation, cellular glucose uptake, or activity of individual compounds at achievable exposure levels. The extent to which modulation of this pathway contributes to the glucose-lowering effects observed in vivo remains to be established.

In streptozotocin-induced diabetic rats, oral administration of the dichloromethane fraction at 10 mg/kg for 45 days was associated with fasting glucose of 116 mg/dL compared with 228 mg/dL before treatment; glycated hemoglobin was 8.4%, compared with 12.9% in diabetic controls [48]. These descriptive values concern a defined fraction in an animal model; they do not establish efficacy in patients.

### 4.6. Cardioprotective Activity

The potential cardiovascular effects of *Kalanchoe pinnata* have been investigated primarily in experimental models, including studies assessing blood pressure, vascular function, heart rate, and biochemical markers of myocardial injury [24,31]. Antioxidant and anti-inflammatory mechanisms have also been proposed to contribute to some of these experimentally observed effects [24,26]. However, evidence supporting clinically relevant cardioprotective effects in humans remains limited.

In experimental models, aqueous *K. pinnata* extracts have been associated with reductions in systolic and diastolic blood pressure [22,25,26]. In a high-salt-induced hypertension model in rats, administration of an aqueous *K. pinnata* extract produced antihypertensive effects [26]. Although these findings support cardiovascular activity under experimental conditions, they are derived from animal studies and do not establish antihypertensive efficacy in humans.

### 4.7. Hepatoprotective and Nephroprotective Activities

The potential hepatoprotective and nephroprotective effects of *Kalanchoe pinnata* have been investigated predominantly in preclinical models of chemically induced organ injury. Experimental evidence indicates that preparations obtained from the plant may attenuate biochemical and histopathological alterations associated with hepatic and renal damage. However, the available evidence remains primarily limited to animal studies, and clinically relevant protective effects in humans have not yet been established [34,49,50].

Hepatoprotective activity has been demonstrated particularly in experimental models of carbon tetrachloride (CCl_4_)-induced liver injury [34,50]. Fresh leaf juice and an ethanolic extract of *K. pinnata* demonstrated hepatoprotective effects in rats based on biochemical, in vitro, and histopathological assessments, with the leaf juice showing greater activity than the ethanolic extract [50]. More recent experimental evidence showed that administration of a *Kalanchoe pinnata*-based beverage to Wistar rats exposed to CCl_4_ significantly reduced serum AST, ALT, ALP, and bilirubin concentrations. Protective effects on these biochemical markers were also observed in a gentamicin-induced hepatotoxicity model [34]. These findings support a potential hepatoprotective effect under experimentally induced toxic injury, although differences in plant preparations, extraction procedures, doses, and experimental models limit direct comparison between studies [34,50].

In a gentamicin-induced nephrotoxicity model, Wistar rats received aqueous *Kalanchoe pinnata* leaf extract at 125 mg/kg/day intraperitoneally alongside gentamicin at 100 mg/kg/day intraperitoneally for eight days, with six rats per group [49]. Biochemical and histopathological renal injury was attenuated relative to gentamicin treatment alone. This parenteral experiment cannot establish benefit or safety for oral or topical human preparations, and concomitant chemical radical-scavenging activity does not prove the mechanism of renal protection.

### 4.8. Applications in Gynecology and Urology

Clinical use in preterm labour concerns defined *Bryophyllum pinnatum* preparations, the synonym under which *Kalanchoe pinnata* is commonly studied in obstetrics [13]. In a retrospective matched-pair comparison of 67 women per group, intravenous *B. pinnatum* was associated with similar pregnancy prolongation to β-agonists, while palpitations or dyspnoea were reported in 34.3% versus 55.2% (*p* = 0.02) [52]. Retrospective matching does not establish treatment equivalence. Two subsequent randomized trials of different *Bryophyllum pinnatum* preparations were discontinued early because of recruitment difficulties, preventing reliable conclusions about efficacy [53]. These findings do not support replacement of established tocolytic treatment. The preparations, designs, and limitations of the human studies are distinguished in Table 3.

The evidence for overactive bladder concerns a manufactured *B. pinnatum* leaf-press-juice preparation, not an unspecified extract or the plant as a whole. Betschart et al. randomized 20 postmenopausal women to Bryophyllum 50% (350 mg dosage units; two units three times daily) or lactose placebo for eight weeks [54]. Daily micturition frequency decreased from 9.5 ± 2.2 to 7.8 ± 1.2 in the active group and from 9.3 ± 1.8 to 9.1 ± 1.6 with placebo, but the primary between-group comparison did not reach statistical significance (*p* = 0.064); quality-of-life improvement did not differ between groups [54]. A later uncontrolled prospective study treated 49 women with idiopathic overactive bladder and nocturia using Bryophyllum 50% chewable tablets (Weleda AG; 350 mg, two tablets with the evening meal and two at bedtime) for three weeks [55]. Nocturnal voids decreased from 3.2 ± 1.4 to 2.3 ± 1.3 (*p* < 0.001), but the absence of a control group precludes causal attribution [55]. These findings apply to the investigated leaf-press-juice products; the “50%” product designation does not indicate 50% flavonoids or bufadienolides.

### 4.9. Experimental Cytotoxic and Antiproliferative Activity

The potential anticancer effects of *Kalanchoe pinnata* have been investigated primarily in preclinical models, with particular attention to bufadienolides such as bryophyllin A, bryophyllin B, and bersaldegenin-1,3,5-orthoacetate [4,14,46]. Experimental studies have associated these compounds with inhibition of cancer cell proliferation and induction of programmed cell death [6,24]. However, the available evidence is predominantly derived from in vitro studies and does not establish anticancer efficacy in humans.

Cell-cycle changes must be attributed to the species and preparation directly tested. In the comparative study of leaf-derived fractions [29], follow-up cell-cycle and mitochondrial experiments used *Kalanchoe blossfeldiana*. Those observations are not direct evidence that *K. pinnata* fractions modulate the same pathways. Likewise, mechanisms reported for isolated bufadienolides do not automatically describe the action of an uncharacterized crude extract.

Normal-cell comparators are essential for interpreting experimental cytotoxicity. Pereira et al. tested *Kalanchoe pinnata* leaf preparations for 24 h against malignant cells and human foreskin fibroblasts (HFFs), using cisplatin as a positive control [75]. For the hot-water preparation, IC_50_ was 10.6 ± 2.5 µg/mL in HeLa cells and 89.5 ± 3.7 µg/mL in HFF. Their ratio is approximately 8.4 (calculated from the reported mean values), a model-specific selectivity estimate rather than a therapeutic index. Corresponding cold-water values were 52.1 ± 5.2 and 90.2 ± 4.1 µg/mL, illustrating preparation dependence. These results neither establish tumour efficacy in vivo nor identify bufadienolides as the active agents in these extracts.

Taken together, the available studies indicate that *K. pinnata* extracts and isolated constituents affect several biological processes, including tissue repair, inflammatory and oxidative responses, glucose metabolism, smooth muscle contractility, microbial growth, and cell proliferation [4,7,24,25]. However, the strength of evidence differs substantially among these areas. Most mechanistic and pharmacological findings originate from in vitro experiments and animal models, whereas human evidence is limited to a relatively small number of studies, including investigations of diabetic foot ulcers, tocolysis, bladder dysfunction, sleep problems, and anxiety symptoms [52,53,54,55,68,76,77,78,79,80,81]. Interpretation of the therapeutic potential of *K. pinnata* therefore requires consideration of the experimental model, preparation and extraction method, phytochemical composition, administered dose, and level of clinical validation [13,24].

Table 2 presents illustrative quantitative experimental findings with their preparation, model, exposure, controls where verified, and principal limitations. Selection is purposive within this narrative review, not a comprehensive comparison of effectiveness. Threshold values and null findings are retained where available. Missing sample sizes, variability measures, or statistical details are not inferred from point estimates, and the table must not be interpreted as a ranking of efficacy or formal assessment of study quality.

### 4.10. Human Studies and Clinical Interpretation

Table 3 distinguishes interventional and observational human evidence from the much larger preclinical literature. It includes studies of participants receiving *Kalanchoe pinnata*/*Bryophyllum pinnatum* preparations; experiments on cultured human cells or excised tissues are not classified as clinical evidence. Preparations vary between fresh leaves, aqueous decoctions, intravenous formulations, tinctures, and manufactured leaf-press-juice tablets, so efficacy and tolerability cannot be pooled across products without considering their composition and route of administration.

In prospective observational studies, subjective sleep outcomes improved during treatment in pregnant women and in patients with cancer [76,77]. These were studies of sleep symptoms, not evidence of anticancer activity. A small restless-legs case series showed heterogeneous subjective and polysomnographic responses [78], and a five-patient dysmenorrhoea series reported reduced pain and analgesic use [79]. These uncontrolled observations are hypothesis-generating. In a single-arm study of anxiety symptoms, 54 of 99 recruited patients provided complete data; the mean Beck Anxiety Inventory score decreased from 27.4 to 22.4 after two weeks and to 20.6 after three weeks, but attrition and the absence of a comparator prevent causal inference [80].

More recent randomized evidence does not establish efficacy for anxiety associated with preterm labour. A 2026 double-blind placebo-controlled pilot randomized 16 women after initial tocolysis, with 10 completing the study; anxiety and sleep scores changed little, with no apparent between-group differences [81]. Early discontinuation and the very small analyzed sample preclude reliable efficacy estimates. Across the human literature, short-term tolerability has generally been acceptable in the investigated settings, but the samples and follow-up are insufficient to exclude uncommon, reproductive, cardiac, or long-term adverse effects. Larger controlled trials with predefined endpoints and chemically characterized products are needed.

Chemical standardization is incompletely reported across these clinical investigations: the “50%” product label specifies a formulation, not an assay of individual flavonoids or bufadienolides in the administered batch. Batch-specific concentrations should not be assumed from manufacturer or nonclinical studies. Outcome labels in this table are not retrospectively assigned as registered primary or secondary endpoints where the original hierarchy could not be verified. Missing regimen details and unreported adverse-event surveillance remain limitations, not evidence of absence of harm.

## 5. Safety and Research Limitations

Safety is an important consideration in the evaluation of *Kalanchoe pinnata* preparations, particularly because their phytochemical composition may include pharmacologically active bufadienolides [23,24,35]. The toxicological profile may vary according to the type of preparation, administered dose, route and duration of exposure, and concentration of individual constituents [4,24]. This variability emphasizes the importance of phytochemical characterization and standardization when interpreting both biological activity and safety data obtained for different *Kalanchoe pinnata* preparations [4,24,63].

### 5.1. Toxicity

Toxicity must be interpreted separately for each *Kalanchoe pinnata* preparation and route. In the study by Harlalka et al., the tested material was a cold-macerated aqueous leaf extract; the reported acute intraperitoneal LD_50_ in mice was approximately 1.8 g/kg [49]. This lethality estimate is not an oral NOAEL and does not identify a safe repeated human dose. The associated renal experiment used intraperitoneal administration in rats over eight days, rather than an oral safety study. Neither the absence of mortality at a tested dose nor observations from a different solvent extract establish long-term safety of an oral product.

Bhavsar and Chandel evaluated fresh *Kalanchoe pinnata* leaf juice in cultured blood lymphocytes from six healthy men aged 20–25 years [82]. Juice aliquots of 50 and 70 µL were tested after preliminary standardization; the higher aliquot significantly reduced the mitotic index and increased sister chromatid exchanges. These are assay-volume exposures, not concentrations in µg/mL or clinical doses. Other measured endpoints did not change significantly. This cellular experiment does not establish an oral or topical NOAEL, a target-organ toxicity threshold, or safety in treated patients.

Secondary reviews describe reproductive and pulmonary findings after exposure to *Kalanchoe pinnata* preparations [3,6,24]. Without verified primary information on plant authentication, extraction, dose, route, treatment duration, and histological assessment, these reports are retained only as safety signals requiring investigation. No quantitative NOAEL or LOAEL is derived from them. Preparation-specific repeated-dose studies, including reproductive, cardiac, pulmonary, hepatic, and renal endpoints, are needed before defining exposure limits.

### 5.2. Bufadienolides as a Potential Source of Adverse Effects

Bufadienolides are particularly relevant to the safety profile of *Kalanchoe pinnata* because this class of steroidal compounds can inhibit Na^+^/K^+^-ATPase, a pharmacological mechanism shared with cardiac glycosides [8,24,31]. Inhibition of Na^+^/K^+^-ATPase alters transmembrane ion gradients and can indirectly increase intracellular Ca^2+^ concentrations through Na^+^/Ca^2+^ exchange, thereby increasing myocardial contractility [24,29,47]. However, excessive exposure to bufadienolides is associated with cardiotoxicity, and cardiac conduction disturbances and ventricular arrhythmias have been reported for compounds belonging to this class [24,29,47]. These findings support caution when considering the safety of bufadienolide-containing *Kalanchoe pinnata* preparations, although such effects should not be interpreted as evidence of equivalent toxicity following typical human exposure to the plant.

Evidence from other bufadienolide-containing *Kalanchoe* species provides complementary toxicological context for this class of compounds [9,13,42]. Livestock poisoning following ingestion of certain *Kalanchoe* species has been associated with krimpsiekte, a syndrome characterized by progressive muscular weakness, paresis or paralysis, and cardiovascular dysfunction [14,42]. Severe poisoning may be fatal [13,18,29]. These observations should not be interpreted as direct evidence of an equivalent toxicological response to *K. pinnata* preparations in humans; rather, they illustrate the potential toxicity of bufadienolides as a pharmacologically active compound class and support the need for species- and preparation-specific safety assessment.

The dose-dependent transition from pharmacological activity to toxicity represents an important consideration for bufadienolide-containing preparations [10,23,47]. Consequently, characterization of bufadienolide content and appropriate standardization are necessary when evaluating the safety of *K. pinnata* preparations [23,57]. This is particularly relevant for repeated exposure, because the long-term toxicological profile and potential for accumulation of individual *K. pinnata* bufadienolides in humans have not been sufficiently characterized.

### 5.3. Limitations of Current Research

Although *Kalanchoe pinnata* has been investigated in numerous experimental studies, the current evidence base has several important limitations [14,22]. Most available data originate from in vitro studies and animal models, while the number of human studies remains limited [14,24,28,47]. These preclinical models are useful for identifying biological mechanisms and potential pharmacological effects but cannot by themselves establish clinical efficacy or long-term safety. Human studies identified in the reviewed literature have focused mainly on selected applications in gynecology and urology, with several studies originating from Switzerland [13,22]. For other proposed applications, including diabetes, cardiovascular disorders, liver disease, and cancer, well-designed randomized clinical trials remain lacking [6,22].

Another major limitation is the lack of standardized *K. pinnata* preparations [6,22]. Extracts used across individual studies differ in plant material, extraction procedure, solvent composition, and phytochemical profile, which complicates direct comparison of results and interpretation of administered doses of active constituents [6,24]. In addition, the concentrations of flavonoids and bufadienolides may vary according to geographical origin, leaf age, and environmental conditions such as light exposure and water availability [10,24,58,60]. These sources of variability make reproducibility and dose standardization difficult.

Potential interactions involving *Kalanchoe pinnata* remain insufficiently characterized. Comparative findings from *Kalanchoe crenata* cannot establish CYP inhibition or a clinical interaction for *K. pinnata*. In particular, the study of *Kalanchoe crenata* with losartan [83] does not serve as species-specific evidence of CYP3A4 or CYP2C19 inhibition by *K. pinnata*. Such extrapolations remain hypothesis-generating and require dedicated experiments with chemically characterized preparations and clinically relevant exposure.

The pharmacokinetic properties of the major secondary metabolites of *K. pinnata* also remain insufficiently characterized [33]. Detailed information on their absorption, distribution, metabolism, and excretion in humans is limited [11,33]. This lack of pharmacokinetic data makes it difficult to define exposure levels, evaluate the possibility of accumulation during repeated use, and establish evidence-based dosing and safety recommendations [22,33].

Overall, interpretation of the therapeutic relevance of *K. pinnata* is currently limited by the predominance of preclinical evidence, variability in extract composition, incomplete pharmacokinetic characterization, and unresolved safety concerns [4,24]. Particular attention should be given to bufadienolide content, possible cytotoxic and genotoxic effects at higher exposures, reproductive toxicity observed in animal models, and the potential for herb–drug interactions [6,24]. Further studies using standardized preparations, clearly defined doses, pharmacokinetic assessment, and appropriately designed clinical trials are required before reliable conclusions regarding efficacy and safety in humans can be drawn.

### 5.4. Comparative Evidence from Other Kalanchoe Species

Studies of *Kalanchoe tomentosa* report isolated β-sitosterol [65]; *Kalanchoe fedtschenkoi* has its own extract profiles and biological assays [66]; and ESKAPE-pathogen testing has involved *Kalanchoe mortagei* and *Kalanchoe fedtschenkoi* [84]. Other cited work concerns wound healing with *Kalanchoe petitiana* [85], antimicrobial effects of *Kalanchoe brasiliensis* [86], cytotoxicity of *Kalanchoe flammea* [87], losartan co-treatment with *Kalanchoe crenata* [83], and murine experiments with *Kalanchoe integra* var. *crenata* [51]. These publications illustrate interspecies differences and are not evidence of corresponding effects, doses, or safety thresholds for *Kalanchoe pinnata*.

## 6. Discussion

The evidence reviewed in this article shows substantial differences in the level of support for the biological effects attributed to *Kalanchoe pinnata*. Wound healing, inflammatory responses, and antioxidant activity have been investigated in multiple experimental models, whereas evidence for several systemic applications, including cardiovascular, hepatic, renal, metabolic, and anticancer effects, remains predominantly preclinical. This distinction is important because the detection of biological activity in cell-based or animal models does not necessarily predict therapeutic efficacy in humans. The phytochemical diversity of *Kalanchoe pinnata* means that extract-level effects cannot be assigned to a particular constituent solely because it is detected in the preparation. Direct isolated-compound experiments support specific activities of QAR [37,44], whereas compositional correlations and in silico target predictions remain hypotheses about the contributors to complex-extract effects.

Mechanistic evidence has different inferential strengths. Direct biochemical inhibition concerns an assayed enzyme; cellular pathway changes require measurements in treated cells; animal biomarkers describe a response in a whole-organism model. An association between improved outcomes and a biomarker does not establish causation. Network-pharmacology predictions and docking scores nominate targets but do not demonstrate target engagement. This distinction is particularly relevant to the computational PI3K/Akt-related predictions [74] and to the transfer of isolated-compound mechanisms to crude extracts.

A major obstacle to comparison between studies is the heterogeneity of the investigated preparations. Differences in geographical origin, plant age, cultivation conditions, extraction solvent, extraction procedure, and phytochemical composition can produce preparations with substantially different concentrations of active constituents. Consequently, apparently conflicting pharmacological results may reflect differences in the tested material rather than true disagreement between studies. This problem is particularly relevant when results obtained with isolated compounds are discussed together with those obtained using crude extracts. The contribution of interactions among individual constituents to the biological activity of complex *K. pinnata* preparations has not been adequately established, and the available evidence does not allow for these interactions to be classified as additive, synergistic, or antagonistic.

Safety represents an additional limitation in translating experimental findings into therapeutic applications. Bufadienolides are of particular concern because their pharmacological activity is accompanied by the potential for cardiac toxicity, while the long-term exposure profile of individual *K. pinnata* constituents in humans remains poorly characterized. Experimental findings concerning cytotoxicity, genotoxicity, reproductive effects, and possible herb–drug interactions further indicate that biological activity should not be interpreted independently of toxicological evidence. This is particularly relevant when preparations differ substantially in their bufadienolide content and overall phytochemical composition.

Cytotoxic concentrations must also be considered against the Na^+^/K^+^-ATPase inhibition and potential cardiotoxicity associated with bufadienolides [47]. The HFF-to-HeLa ratio for a leaf preparation [75] does not measure cardiac safety; fibroblasts are not a substitute for cardiomyocyte electrophysiology or systemic toxicokinetics. Because matched cytotoxicity, bufadienolide concentration, achievable human exposure, and cardiac toxicity data are lacking for the same preparations, a clinically useful therapeutic window cannot be established.

### Future Research Directions

Genetically modified *Kalanchoe pinnata* expressing cecropin P1 represents a distinct experimental platform for future research. Studies of transgenic preparations reported antibacterial and antifungal effects in infected animal-wound models [36,38]. These results concern a recombinant antimicrobial peptide delivered in a plant-derived preparation and must not be presented as clinical evidence or as activity of native *Kalanchoe pinnata* constituents. Further work should establish reproducible peptide expression and dose, comparative activity against nontransgenic preparations, product composition, and safety before clinical translation [36,38].

Future studies should prioritize the use of chemically characterized and standardized preparations, with explicit reporting of plant origin, extraction procedure, concentrations of major constituents, and administered doses. Pharmacokinetic studies are also needed to determine the systemic exposure, metabolism, and elimination of major flavonoids and bufadienolides. For biological effects currently supported mainly by preclinical evidence, appropriately controlled human studies will be necessary to determine whether the observed experimental effects are clinically reproducible. Particular attention should be given to dose–response relationships, long-term safety, and the identification of preparations in which potential biological benefits can be evaluated against clearly defined toxicological risks.

## 7. Conclusions

The available literature confirms that *Kalanchoe pinnata* contains a chemically diverse group of secondary metabolites, particularly flavonoids, bufadienolides, phenolic acids, and triterpenes, several of which have demonstrated biological activity in experimental studies. The most frequently investigated effects involve wound healing, inflammatory and oxidative processes, microbial growth, glucose metabolism, and cancer cell proliferation. However, the strength of evidence differs substantially among these areas, and most reported effects remain supported primarily by in vitro experiments and animal models rather than controlled human studies.

Interpretation of these findings is further complicated by substantial variability in plant material, extraction procedures, and phytochemical composition, which limits direct comparison between studies and makes dose standardization difficult. Pharmacokinetic data for the principal bioactive constituents also remain limited. In addition, the presence of pharmacologically active bufadienolides, together with experimental findings concerning cytotoxicity, genotoxicity, reproductive effects, and cardiovascular responses, highlights the need for careful safety assessment of *Kalanchoe pinnata* preparations intended for human use.

Further research should prioritize chemically characterized and standardized preparations, clearly defined dose–response relationships, pharmacokinetic characterization, and long-term toxicological assessment. For proposed therapeutic applications currently supported mainly by preclinical evidence, well-designed controlled clinical trials are required to determine whether the reported biological effects are reproducible and clinically relevant in humans. Until such evidence becomes available, the therapeutic significance of *K. pinnata* should be interpreted cautiously and in relation to the specific preparation, experimental model, administered dose, and associated safety profile.

## Figures and Tables

**Figure 1:**
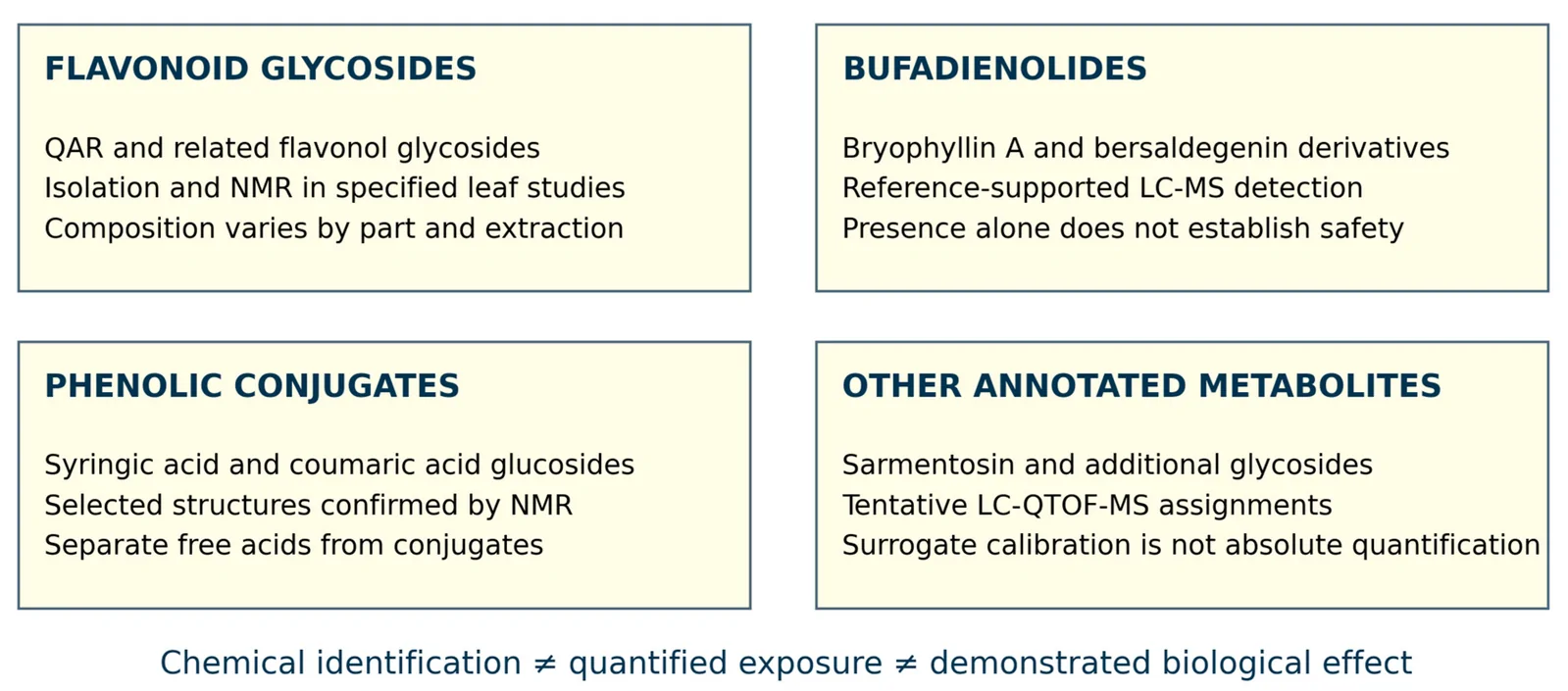
Representative phytochemical evidence for *Kalanchoe pinnata*. Original schematic based on primary analytical studies [29,57,63]. No third-party plant photograph or icons are used. The scheme does not assign a biological effect to an entire chemical class or imply that every constituent occurs in every preparation.

**Table 1:** Representative phytochemical constituents reported in *Kalanchoe pinnata*, their plant sources, extraction procedures, and analytical evidence.

Class and Representative Compounds	Plant Part	Extraction/Fractionation	Analytical Evidence	Ref.
Quercetin glycosides: QAR and related quercetin glycosides	Leaves; primary metabolite-profile study [57]. Origin and voucher not verified in this synthesis.	Methanol extract; chromatographic isolation	Isolated flavonoids characterized by 1H and 2D NMR; structural identification distinguished from quantification; absolute concentration not established here.	[57]
Kaempferol glycosides, including the arabinose–rhamnose diglycoside (kapinnatoside)	Leaves; primary metabolite-profile study [57]. Origin and voucher not verified in this synthesis.	Methanol extract; chromatographic isolation	NMR-supported structural identification within the isolated flavonoid series; absolute concentration not established here.	[57]
Other flavonoids: myricetin, acacetin and diosmetin glycosides	Leaves; primary metabolite-profile study [57]. Origin and voucher not verified in this synthesis.	Methanol extract; chromatographic isolation	NMR-supported structures; no universal abundance ranking inferred; absolute concentration not established here.	[57]
Flower flavonoids: QAR and quercetin 3-O-D-glucuronide	Flowers; Villa de Leyva, Boyacá, Colombia; identified by N. Garcia; voucher HPUJ-30749.	Hot-water infusion; centrifugal partition chromatography for marker purification	CPC-purified markers; UV/MS assignments; UPLC-PDA total flavonoids 9.97 ± 0.17% in the tested extract, calibrated with rutin. This is not absolute compound-specific quantification.	[63]
Bufadienolides: bryophyllin A; bersaldegenin-1-acetate, -3-acetate and -1,3,5-orthoacetate	Leaves; primary metabolite-profile study [57]. Origin and voucher not verified in this synthesis.	Methanol extract used for metabolite profiling	Chromatographic/MS detection supported by authentic reference compounds; not a claim that all were newly isolated from *K. pinnata*; absolute concentration not established here.	[57]
Quercetin glycosides: QAR and related quercetin glycosides	Leaves; primary metabolite-profile study [57]. Origin and voucher not verified in this synthesis.	Methanol extract; chromatographic isolation	Isolated flavonoids characterized by 1H and 2D NMR; structural identification distinguished from quantification; absolute concentration not established here.	[57]
Phenolic glucosides: syringic acid β-D-glucopyranosyl ester and 4′-O-β-D-glucopyranosyl-cis-p-coumaric acid	Leaves; primary metabolite-profile study [57]. Origin and voucher not verified in this synthesis.	Methanol extract; chromatographic isolation	Isolated compounds with 1H and 2D NMR structural confirmation; absolute concentration not established here.	[57]
Phenylpropanoid and benzoic-acid derivatives; acyclic alcohol glycosides; sarmentosin annotation	Fresh leaves; commercial garden source, Gdańsk, Poland; voucher 21762, GDMA Herbarium.	95% ethanol extraction, followed by water/dichloromethane partition; aqueous fraction analyzed	LC-QTOF-MS tentative annotations; semi-quantitation with surrogate standards. Compound-specific absolute concentrations not established.	[29]

Abbreviations: QAR, quercetin 3-*O*-α-L-arabinopyranosyl-(1 → 2)-α-L-rhamnopyranoside; NMR, nuclear magnetic resonance; PDA, photodiode array detection. Extraction solvents are distinguished from solvents used subsequently for analytical sample dissolution or chromatographic elution. Tentative annotations and class-level screening results are not equivalent to isolated, structurally confirmed compounds; no extract-wide mass percentage is inferred from peak areas.

**Table 2:** Selected quantitative findings from experimental studies investigating the biological activity of *Kalanchoe pinnata* preparations and isolated constituents.

Biological Activity	Preparation/Compound	Experimental Model	Main Quantitative Finding	Controls and Limitations
Anti-inflammatory	QAR/KPFV	In vitro COX inhibition assay	COX-1 IC_50_ = 22.1 µg mL^−1^; COX-2 IC_50_ > 50 µg mL^−1^	Ovine COX assay; indomethacin IC_50_ 5.9 and 31.2 µg/mL for COX-1 and COX-2. Enzyme selectivity is not a clinical safety measure.
Antinociceptive	Aqueous flower extract (KPFE)	Mouse acetic-acid writhing test; *n* = 5–6/group; s.c. 30 min before challenge	ID_50_ 164.8 mg/kg; 300 mg/kg reduced writhing by 70.1%; 30 mg/kg was ineffective.	Saline and indomethacin 10 mg/kg controls; means ± SEM; ANOVA with Dunnett comparison. Animal pain-response endpoint.
Wound healing	6% aqueous leaf extract cream	Rat excision wound model	95.3 ± 1.2% wound healing on day 12	Negative-control comparison reported; point estimate retained with reported variability. Sample size and complete statistical analysis not verified.
Wound healing	0.15% purified QAR cream	Rat excision wound model	97.5 ± 0.8% wound healing on day 12	Reference neomycin/bacitracin preparation: 96.7 ± 0.8%. Similar means do not establish equivalence; sample size not verified.
Antioxidant	Aqueous leaf extract (cold maceration)	DPPH assay; 0.1 mM methanolic DPPH; 30 min at 25 °C; 517 nm	EC_50_ = 116.25 µg mL^−1^	Ascorbic acid EC_50_ 11.25 µg/mL in the same assay. Cell-free chemical capacity; no clinical inference.
Antidiabetic	Methanolic leaf extract	α-Amylase inhibition assay	IC_50_ = 29.50 ± 0.04 µg mL^−1^	Cell-free inhibition; not evidence of predicted pathway modulation. Comparator and replicate details not verified.
Antidiabetic	Methanolic leaf extract	α-Glucosidase inhibition assay	IC_50_ = 32.04 ± 0.35 µg mL^−1^	Cell-free inhibition; no demonstration of glycemic efficacy. Comparator and replicate details not verified.
Antidiabetic	DCM fraction of leaf steam distillate	Streptozotocin-induced diabetic rats	Fasting glucose 228 → 116 mg/dL; oral 10 mg/kg for 45 days.	Diabetic-rat model; no human efficacy inference. Dispersion and exact group size not verified.
Antidiabetic	DCM fraction	In vitro insulin secretion assay	Test concentration: 10 µg/mL. A 3.29-fold increase versus a positive control is reported in the primary abstract; full comparator details remain unverified.	Fraction-level secretion response; no direct channel-binding evidence. Full assay statistics and replicate count not verified; no claim of drug superiority.
Antimicrobial	Water fraction of ethanolic leaf extract	*C. albicans* ATCC 10231; broth microdilution	MIC > 2000 µg/mL; no inhibition within that range	Inactive result; ketoconazole reference; no matched normal-cell SI.
Cytotoxicity	Hot-water leaf preparation	HeLa and nonmalignant HFF; 24 h	IC_50_ 10.6 ± 2.5 vs. 89.5 ± 3.7 µg/mL	Cisplatin control; calculated mean-value ratio ≈ 8.4; not a systemic therapeutic index.

**Table 3:** Human studies of *Kalanchoe pinnata* (*Bryophyllum pinnatum*) preparations: design, exposure, clinical findings, and limitations.

Study/Indication	Design and Sample	Preparation, Dose and Duration	Main Findings	Limitations/Tolerability	Study/Indication
Torres-Santos et al., 2003 [73] Cutaneous leishmaniasis	Single case; one 36-year-old man	Oral fresh leaves, 30 g wet weight/day for 14 days	Lesion stopped growing and decreased slightly; complete cure not established	Uncontrolled case; no adverse reactions reported during brief exposure; insufficient evidence of efficacy or general safety	Torres-Santos et al., 2003 [73] Cutaneous leishmaniasis
Plangger et al., 2006 [52] Preterm labour	Retrospective matched pairs; 67 women per group	Intravenous *B. pinnatum* leaf-derived preparation versus β-agonists; treatment according to clinical practice	Pregnancy prolongation 6.2 versus 5.4 days (not significant); palpitations/dyspnoea 34.3% versus 55.2% (*p* = 0.02)	Nonrandomized comparison; confounding remains; similar outcomes do not establish noninferiority	Plangger et al., 2006 [52] Preterm labour
Betschart et al., 2013 [54] Overactive bladder	Double-blind placebo-controlled RCT; 20 postmenopausal women (10/group)	Bryophyllum 50%, 350 mg units; two units three times daily for 8 weeks; lactose placebo	Voids/24 h: 9.5 → 7.8 versus 9.3 → 9.1; primary comparison *p* = 0.064; no between-group QoL benefit	Pilot size; primary endpoint not significant; adverse-event frequency similar, no serious events	Betschart et al., 2013 [54] Overactive bladder
Lambrigger-Steiner et al., 2014 [76] Sleep problems in pregnancy	Prospective multicentre observational study; 49 women	Weleda leaf-press-juice tablets, 350 mg/Bryophyllum 50%; physician-selected dose; 2 weeks	Fewer awakenings and improved subjective sleep quality; reduced daytime sleepiness; no demonstrated improvement in sleep duration, latency or fatigue	No control group; subjective endpoints and short follow-up; no serious adverse drug reactions detected	Lambrigger-Steiner et al., 2014 [76] Sleep problems in pregnancy
Simões-Wüst et al., 2015 [77] Sleep problems in cancer patients	Prospective observational study; 28 recruited, 20 analyzed	Bryophyllum 50%, 350 mg tablets; most often two with the evening meal and two at bedtime; 3 weeks	PSQI: 12.2 ± 3.62 → 9.1 ± 3.61 (*p* < 0.01); ESS: 8.4 → 7.1 (*p* < 0.05); fatigue unchanged	No control; attrition and concomitant treatment; six patients reported possibly related discomfort; no serious reactions; not a cancer-treatment trial	Simões-Wüst et al., 2015 [77] Sleep problems in cancer patients
Simões-Wüst et al., 2018 [53,79] Preterm-labour prophylaxis (trial I)	Double-blind placebo-controlled randomized trial; *n* = 26, as summarized in [79]	*B. pinnatum* 33% ethanolic tincture versus placebo; prophylactic regimen	Discontinued early because of slow recruitment; insufficient data to establish efficacy	Under-recruitment; not evidence of benefit or absence of benefit; tolerability described as good	Simões-Wüst et al., 2018 [53,79] Preterm-labour prophylaxis (trial I)
Simões-Wüst et al., 2018 [53,79] Acute tocolysis (trial II)	Open-label randomized comparison with nifedipine; *n* = 27, as summarized in [79]	Bryophyllum 50% leaf-press-juice tablets versus nifedipine; acute tocolysis regimen	Discontinued early; clinical efficacy could not be established conclusively	Open-label design and small sample; no reliable noninferiority conclusion; tolerability described as good	Simões-Wüst et al., 2018 [53,79] Acute tocolysis (trial II)
von Manitius et al., 2019 [78] Restless legs syndrome	Consecutive case series; five patients; polysomnography before/after	Mainly Bryophyllum 50% tablets (individual regimens, e.g., 4–6/day); one patient received 33% tincture; about 2 months	Some subjective and polysomnographic improvements; responses were heterogeneous, including a patient without major sleep/RLS improvement	No control; different formulations and concomitant drugs; cannot establish a treatment effect	von Manitius et al., 2019 [78] Restless legs syndrome
Mirzayeva et al., 2023 [55] Nocturia with overactive bladder	Multicentre nonrandomized prospective study; 49 women	Weleda Bryophyllum 50%, 350 mg chewable tablets; two with evening meal and two at bedtime; 3 weeks	Nocturnal voids: 3.2 ± 1.4 → 2.3 ± 1.3 (*p* < 0.001); PSQI: 7.7 ± 3.7 → 6.6 ± 3.4 (*p* = 0.004)	No comparator; short duration; no serious adverse drug reactions reported	Mirzayeva et al., 2023 [55] Nocturia with overactive bladder
Zurfluh et al., 2023 [79] Dysmenorrhoea	Case series; five patients aged 14–27 years	Weleda Bryophyllum 50%, 350 mg chewable tablets; six/day in individualized schedules; follow-up 3–6 months	All five reported ≥ 2-point pain-score reduction; four reduced analgesic use	No control; very small sample and concomitant analgesics; no adverse reactions reported	Zurfluh et al., 2023 [79] Dysmenorrhoea
Farida et al., 2024 [68] Diabetic foot ulcers	Quasi-experimental pretest–post-test control study; 40 patients (20/group)	Topical aqueous decoction: 100 g leaves/1 L water, boiled to 500 mL; six cleansings/14 days; 0.9% saline comparator	Both groups improved; authors reported greater BWAT improvement with leaves (*p* = 0.003)	Baseline group differences; unclear indicator-level statistical reporting; short follow-up; detailed adverse-event assessment not reported	Farida et al., 2024 [68] Diabetic foot ulcers
Huber et al., 2024 [80] Anxiety symptoms	Prospective single-arm study; 99 recruited, 54 complete datasets	Weleda Bryophyllum 50%, 350 mg chewable tablets; two three times daily; 3 weeks	BAI: 27.4 ± 12.0 → 22.4 ± 12.1 at week 2 and 20.6 ± 12.9 at week 3 (both *p* < 0.001)	No control; substantial attrition; causality not established; good reported tolerability	Huber et al., 2024 [80] Anxiety symptoms
Zurfluh et al., 2026 [81] Anxiety related to preterm labour	Double-blind placebo-controlled pilot RCT; 16 randomized, 10 completed (5/group)	Weleda Bryophyllum 50%, 350 mg chewable tablets; two four times daily for 14 days after initial standard tocolysis	Anxiety/sleep scores changed minimally; no apparent between-group differences; 9/10 completers delivered at term	Early discontinuation and very small sample; selection/measurement limitations; no reliable efficacy or long-term safety conclusion	Zurfluh et al., 2026 [81] Anxiety related to preterm labour

Abbreviations: BAI, Beck Anxiety Inventory; BWAT, Bates–Jensen Wound Assessment Tool; ESS, Epworth Sleepiness Scale; PSQI, Pittsburgh Sleep Quality Index; QoL, quality of life; RCT, randomized controlled trial; RLS, restless legs syndrome. Arrows indicate within-group baseline-to-follow-up changes, which do not establish superiority over a comparator. The designation Bryophyllum 50% describes a leaf-press-juice product, not a 50% concentration of an isolated active compound. Study-specific regimen details are given where available; the 2018 trial sample/formulation summaries are explicitly cross-referenced to [79]. This table is a narrative synthesis rather than a formal risk-of-bias assessment.

## Data Availability

No new data were created or analyzed in this study. Data sharing is not applicable to this article.
